# Collectanea Medica, Consisting of Anecdotes, Facts, Extracts, Illustrations, Queries, Suggestions, &c.

**Published:** 1816-10

**Authors:** 


### COLLECTANEA MEDICA,

CONSISTING OF

### ANECDOTES, FACTS, EXTRACTS, ILLUSTRATIONS, QUERIES, SUGGESTIONS, &c.

RELATING TO THE

*History or the Art of Medicine, and the Auxiliary Sciences.*

Quicquid agunt medici,
nostri farrago libelli.

IN our last number, we expressed a wish of arresting the attention of our readers to the bold operation proposed, and, as far as we can candidly conclude, successfully attempted by Professor Osiander. Our particular intention was, that the disease usually called Cancer Uteri should be properly distinguished from the true Cancer of the Breast; because, for the latter, we are free to acknowledge that we have found the operation so frequently fail as to render us very cautious in recommending it.

It appears, that so long ago as the days of Aretæus, that valuable writer made a distinction between the malignant ulcer of the uterus and true carcinoma.* Among the moderns the distinction was lost, till Mr. Hunter revived it. As this was not done in any of his published works, we shall take the liberty of tracing the progress of the enquiry as far as it has appeared in print. We are the more induced to do this, because the first edition of "Adam's on Morbid Poisons" is now rarely met with, and the passage to which we refer, is neither contained in the second edition, nor in his Treatise on the Cancerous Breast. The following is the introductory part.

"There is only one other cancer with which I shall detain the reader, which, from its frequency and constant fatality, may well arrest a medical writer in his career. Of all the calamities to which a sex that seems destined to support the largest share of human misery is obnoxious, the cancerous uterus is the heaviest. This will not, I apprehend, be disputed; whether we consider the situation of the disease, almost any one of its attendant symptoms,

* De causis & sidib. morb. Diuturn. lib. 2, cap. 11, p. 64. Edit. Lugduni Batav. 1735.

or or the certainty of a life more wretchedly protracted than by any means that the ingenuity of Indian torments can boast. We are ready enough to call the gout *opprobrium medicorum*, because we know, in most instances, it may be called *opprobrium divitiarum*. But what shall we say of a disease that is every day occurring, and the medical progress of which has hitherto extended no further than to confound it with another, which it only resembles in being painful and incurable; and to apply remedies which have derived their reputation from curing diseases different from either! The latter part of this proposition is, I trust, already proved. If the former rested on my own authority, I might have only ventured to ask, where are the cists, where the fungus, where the increased size, in the most common of all the cases of cancerous uterus. I might ask, do we discover more than a sloughing phagedænic ulcer, with thickened hard lips, which slowly destroys the organ and all the neighbouring parts, till *the kindest of physicians* puts a period to the disease and the patient's sufferings.

"That true carcinoma may attack the uterus as well as any other part, is a position I shall not attempt to deny; but that the above, the most common species of what is called cancerous uterus is improperly confounded with the disease in the breast, is a remark for which I was first indebted to Mr. Hunter. A writer,* whom he could only know by name, but with whose works every physician practising in London must be well acquainted, takes some pains to correct this error, which existed in his time; and Morgagni describes two cases, in one of which this ulcer had committed its usual ravages on the cervix, in the other it had extended to the neighbouring parts, while the rest of the uterus was sound.†

But neither Hunter, Aretæus, or Morgagni, is any authority for a plain matter of fact, on which any one may satisfy himself. It must, therefore, depend on the observations of those who have the best opportunities of seeing such appearances. From the general accuracy of Dr. Baillie's descriptions, and from the universality of his enquiries, we might expect a solution of this important difficulty; but his account of the diseases of this important viscus is much too short and confused. If the section of the cancerous uterus does sometimes exhibit an appearance similar to other parts in what is called a scirrhous state, this is neither a necessary nor the most common attendant on the sloughing phagedæna of the cervix and neighbouring parts. I had an opportunity of inspecting in one winter the uteri of four women, who died of the complaint, and none of them exhibited the least appearance of disease a quarter of an inch beyond the ulcer. None of them appeared enlarged, nor was there any 'whitish firm substance, intersected by strong membranous divisions.' In the melancholy collection preserved by Dr. Lowder, few appear enlarged, and of these very few be-

"* Aretæus, cap. xi.

"† De Causis & Sedibus Morborum Epist. xlvii. 8, & xxxix. 33; yond yond the size that this uncertain viscus often exhibits in a sound state."*

This work was published soon after Dr. Baillie's first edition of "Morbid Anatomy." In all the subsequent editions, Dr. B., with a candour which has distinguished his character through life, altered the passage, and added the following note to his alteration.

"This diseased change I formerly confounded with the scirrhous enlargement of the uterus, considering them as varieties of the same disease, and therefore blending their description together; but, in consequence of the accurate observations of Dr. Adams, in his Essay upon Morbid Poisons, I have thought it proper to separate them."

Mr. Clarke, whose work we noticed in a late Number of our Journal, has renewed the subject with equal judgment and modesty.—See London Medical and Physical Journal, page 337, vol. xxxiii.

Having premised thus much, we conceive we have prepared our readers for one of the boldest operations that has been suggested in this age of chirurgical improvement.

"*Observations on the Cure of Cancer of the Womb by Excision*: by F. Bj. Osiander, Professor of Midwifery in the University of Gottingen. †

"Cancer of the womb is one of the most dreadful evils which can afflict the female sex. More frequent in many places than is commonly believed, it commits greater devastations in an insidious but malignant manner, and does more harm than many other diseases,—and has seldom yielded to any external or internal remedies hitherto used.

"The superintendance for many years of the Royal Clinical Hospital of Gottingen having given Professor Osiander frequent opportunities of examining and treating persons suffering under this disease, he, with the active assistance of the young physicians and surgeons who frequented his *clinicum*, employed, in many such cases, all the means, external and internal, which were known or imagined, in order to ascertain by what means, or by what mode of treatment, a stop could be put to the progress of this terrible disease; but he learned from experience the melancholy fact, that little else could be done with it than, in a few cases, to retard its progress, and render the sufferings of the patient more supportable; that is to say, the pain, the bleeding, and the bad smell;—

---

* Adams on Morbid Poisons, first edit. page 177.

"† This paper was communicated by John Thomson, M.D. Professor of Military Surgery in the University of Edinburgh, at the request of the author. It was published in the Göttingische gelehrte Anzeigen so long ago as the 13th of August 1808; but, when the subject is important, and the information new, the date of a communication is of no consequence, except as a historical fact.—*Editor*" [of the Edin. Journal.]

to to effect a cure seemed absolutely impossible. Long before he attempted any operation with the view of curing this disease, he conceived it might be possible to cure cancer of the womb by excision, in the way that has been long known and practised in the case of cancerous mammæ. The possibility of the success of this operation, he founded on the repeated and well known observation, that the inverted and prolapsed uterus has been sometimes cut off designedly by surgeons, and by midwives through ignorance, and yet the persons survived its excision. A woman still lives in the neighbourhood of Gottingen, from whom, twenty-six years ago, an old ignorant midwife cut, or rather sawed off, with a bread knife, the uterus inverted and prolapsed after delivery. The late Professor Wrisberg at that time read the history of this woman before the Royal Society; and it is to be found in this Journal (Götting. gelehrte Anzeigen) for 1787, No. 81, p. 810. It likewise appeared in the eighth volume of the Commentat. Götting.

"These observations induced Professor Osiander to propose, fifteen years ago, in his lectures on the diseases of women, the bold measure of curing cancer of the uterus by extirpation; and he suggested various methods of doing so; among others, the very same which the late Dr. Struve, one of his former pupils, and physician in Prenzlau, afterwards published as his own invention, in the third number of the sixteenth volume of Hufeland's Journal for Practical Medicine, in the year 1803, and against which Professor Osiander cautioned the public at the time. Professor Osiander, however, found the performance of this operation to be very different from what he had conceived, when, at last, on the 5th of May 1801, an opportunity offered itself to perform it. The patient was a widow, whose situation was indeed as deplorable as could be imagined. The vagina was distended by a carcinomatous fungus of the orifice of the womb, as large as a child's head. It was extremely fetid, and bled very violently. The fungus was seized and brought low down in the vagina by means of Smellie's forceps; but, in endeavouring to put a loop round the neck of the womb, the fungus broke off, and the bleeding was terrible. The young physicians and surgeons, and a few experienced physicians who were present, among whom Professor Osiander quotes as a witness Dr. Althof, physician to the Elector of Saxony, advised him to give up the operation, because they believed the woman would not be able to undergo it, after losing so much blood. But the patient herself begged that the operation once begun should be completed, and encouraged the professor to continue it, to the great surprise of all who were present. As there was no longer any of the neck of the womb hanging down in the vagina, by which the uterus could be drawn down, necessity, the mother of many inventions, suggested to Professor Osiander the idea of pulling down the uterus by passing threads through it with a needle, and securing it in this manner till the operation was finished. Crooked needles were immediately mounted with fine waxed pack-thread, and carried with great caution to the bottom of the vagina, pushed through it and through the body of the womb, and then brought out through the inner orifice of the uterus; for the neck of the womb and its external mouth were already destroyed by the cancerous fungus. In this way four threads, one from each side, from before, from behind, and from both sides, were brought through, by which the uterus was gently drawn low down into the vagina, and kept fast as soon as it was near the external orifice. Professor Osiander then introduced a strong Pott's bistoury under the fore-finger of the right hand, and cut horizontally through the womb above the scirrhous part, as straight as if it were divided out of the body, where he could see it. The part that was cut off, preserved in spirits of wine, along with many other products of similar operations, was exhibited to the Royal Society. The bleeding for an instant was violent, but was very soon stopt by a sponge saturated with a powder made of equal parts of alum, gum Arabic, and colophony, applied in the vagina. After the bleeding had ceased, sponges dipt in a solution of sacchar. saturni and vinegar, were applied to mitigate the inflammation, and as soon as matter was perceived on the sponge, suppurants were employed. Professor Osiander for this purpose made use of a mixture of the extract of unripe walnut-shells, with honey, and red precipitate, which was introduced on sponges with great caution, so that the anterior part of the vagina was scarcely touched by it. When the discharge is very copious, the quantity of the mixture must be reduced, and the red precipitate omitted; if inconsiderable, its quantity must be increased. The cure of this first operation went on so rapidly by the assistance of tonics, particularly cinchona, internally, that the patient could leave her bed in the third week, and in the fourth walked about, totally recovered.

"The success of this first operation gave Professor Osiander the courage to undertake it again soon afterwards; and he has now performed it nine times, and always with similar success. Even one of those patients, after enjoying good health for three years, came to him to be operated upon a second time, for the cancer broke out again, and he performed it with the same success as before. He reserves for a future occasion the circumstantial relation of each case, and at present only gives the following result of the observations, which he had so many opportunities of making with regard to this disease, and others which are frequently mistaken for it.

"The scirrhus and cancer of the womb begin almost always on the external orifice of the uterus, and proceed from thence to its body; and often before the disease has destroyed half of the womb, death puts an end to the agonies of the sufferer. In rare cases, an ulcer is formed in the bottom of the womb, which degenerates into a cancer that admits of no cure. But, in the first case, a perfect cure may be obtained by cutting out in time the scirrhous and cancerous portions. When the cancer spreads from the orifice of the womb down into the vagina, which it very often does, no cure is to be expected from excision.

“ True scirrhus and cancer are, however, very often mistaken for other diseases.

“ First, for the benignant swelling and thickening of the womb, which often happens after abortion, miscarriage, or difficult natural labours. The neck of the womb and its orifice swell to twice their natural thickness. The weight and pressure of the whole womb becomes disagreeable to the woman, and causes a sensation as if it would fall out of the body. This swelling is caused by accumulation of blood in and about the womb, from *plethora localis;* and being for the most part joined with hæmorrhoidal symptoms, and indeed oftener with hæmorrhoids in the vagina, physicians and others, even accoucheurs skilled in investigation, are frequently misled, and conceive it to be a bleeding cancer of the womb. This swelling (εξογκωσις) and thickening (σκληρωμα) of the womb may remain during life without becoming malignant, when no other cause comes into operation. It may be cured by remedies which take away the *plethora localis;* but it may also degenerate into a true scirrhus and cancer, when local irritation or morbid matter, *e. g.* a chronic exanthema, repelled from the skin to the womb, supports and augments the swelling.

“ Professor Osiander had repeated opportunities of treating such cases, which, as well as their causes, are frequently misunderstood. In some instances, a round hard pessary, introduced with force, so squeezed and pressed the neck of the womb, as to cause an obstinate scleroma, which could only be cured by an operation. In one case, cancer of the womb, lameness of the lower extremities, and miserable death, were the consequences of a pessary forgotten in the vagina, and pressing on the mouth of the womb.

“ Another cause of scleroma, and several symptoms resembling cancer of the womb, which is often misunderstood, is the incarceration of a retroverted unimpregnated womb. The fold behind the womb is in many women of such a form, that it is very narrow above, and wide below, nay, even sometimes divided into two parts by a perpendicular partition. After labour, and by hard straining to have a stool, the bottom of the womb is sometimes pressed backwards and downwards into this fold, and cannot recover itself again; the womb begins to swell, and causes a great pressure on the anus, irritation on the bladder, and violent hæmorrhoidal symptoms. Professor Osiander met with four cases of this kind, three of which he treated with success, instructed by the first.

“ In the first case, a widow complained of constant hæmorrhoidal symptoms, a disagreeable pressure on the anus, and frequent faintings. The seat of pain was not examined, but external and internal remedies for the hæmorrhoids were administered. She was suddenly attacked by nervous fever, and died. On opening the body, the unimpregnated womb was found retroverted, and adhering to the bottom of the fold. Behind this a wide spreading ulcer was discovered, with traces of inflammation on the intestines and spleen. The ovaria were indurated.

“ In

"In a second case, after abortion, the hæmorrhoidal symptoms continued a long time, and caused excruciating pain, and most obstinate costiveness. On examination, the womb was found retroverted and incarcerated, with a sarcoma on its outer and anterior side, of the size of a very large chesnut. As it was impossible to bring it back by the common taxis with the point of the finger, Professor Osiander contrived a peculiar manner of operating, which, in this, as well as in the two following cases, gave speedy relief. He introduced his dilator from above to the bottom of the retroverted uterus, and turned the instrument round at once. By this method, the bottom of the womb started up suddenly. In another case, the womb, together with a polypus as big as a pear, had been retroverted upwards of a year, and had caused effects like those of pregnancy, hæmorrhoidal affections, and symptoms resembling cancer. Professor Osiander recognized the disease at the first examination; raised up the womb as in the former case; and, after a few days, he dilated the uterus with the same instruments, and cut the polypus out by the root, with crooked-bladed scissars of his own invention, which he is in the habit of doing with all polypi, for he never either ties or tears them out. In the last case, the womb was retroverted for some years, and had caused the severest hæmorrhoidal affections, as well as difficulty in passing urine, and going to stool. These symptoms were aggravated by the many warm injections that were applied *in ano* and *in vagina*, the affection being mistaken by the physicians for a cancer of the womb. But Professor Osiander, on the first examination, discovered the real nature of the disease, and, by the skilful application of his dilator soon put an end to the long existing cause.

"Secondly, Cancer of the womb is often confounded with ulcerated polypus of the womb discharging fetid ichor, and with sarcoma of the uterus. These constitute a peculiar, and not very uncommon disease of the female sex, generally misunderstood, and frequently left as incurable; but Professor Osiander, for some years past, has cut out and cured many, after a peculiar and successful method of his own, and his method of treatment, and his observations on it, will be laid before the public on some future occasion.

"The causes of cancer of the womb are manifold. One very common cause, besides mechanical injury of the external orifice of the womb, is syphilis, obvious or latent, or a scrofulous, herpetic, atrabilious and gouty disposition. Besides, all eruptions of the skin fall readily on the private parts of women, in consequence of a continual local irritation, and cause fluor albus of various kinds, which sometimes precedes cancer of the womb, and sometimes accompanies it.

"Among the internal remedies used in the cure of cancer of the womb, along with the operation, Professor Osiander found none so powerful as mercurials with antimonials, and the free use of diuretic drinks. He has not hitherto ventured to make trial of arsenic, although he believes, that, in doubtful cases, it might be

 cautiously cautiously used; but he has not as yet met with a proper opportunity, which occurs oftener in hospitals than in private practice. Professor Osiander admits only now and then into the lying-in-hospital, of which he is the director, a patient afflicted with cancer of the womb, where there is some hope of her being cured; and last year, he again relieved such a patient from her long sufferings, by operating upon her in the presence of many of his pupils. He never made a secret of his manner of operating, although he has been publicly upbraided for having done so; nay, never was such an operation undertaken without strangers as witnesses. He has taught it every year in his lectures, and often performed it publicly before his pupils. He communicated it to every physician in his own country or abroad, who wrote or spoke to him on the subject. Last year he sent an account of the operation to Mr. Maunoir, senior, of Geneva, in a letter, written in the Latin tongue, which Mr. Maunoir communicated to Mr. Martin, surgeon to the hospital in Lyons, and to the medical faculty of Montpellier. This letter, along with Mr. Maunoir's theory of cancer, was printed in the Annals of the Medical Society of Montpellier. Even very lately Professor Osiander performed the operation in Switzerland, in the presence of three skilful physicians and surgeons. What may be its ultimate success is not yet (1808) known, but hitherto the accounts are very favourable.

" Professor Osiander has two methods of operating for cancer of the womb:

" First, in the manner already described. The patient must be placed on a high labour-stool, or on a table, in the position for the operation for the stone, or for delivery, and held fast. The private parts are cleaned out by injections, and softened with an ointment. The fungus is taken away with the finger, or with an instrument. If the bleeding is great, it will be stopped by applying a sponge dipped in vinegar with astringent powder; if not, the operation is immediately continued. For piercing the uterus, Professor Osiander uses small crooked needles made of soft steel, so that their points can be easily bent. Hard tempered needles are in danger of being broken, and if the broken points be not found, they might do the greatest harm. The greatest difficulty is, to bring the needles through the uterus, till by practice the necessary dexterity is acquired; but the length to which this can be carried, the following circumstance will show. Last year, in the lying-in-hospital here, it happened, in a public operation, that the threads were drawn out of the needles, after they had been pushed through the uterus. Professor Osiander left the needles in the uterus, and threaded them again within the vagina, without requiring the assistance of a contrivance for throwing in light. The operator can and must, in such a case, acquire by practice the same precision and dexterity which many blind people have, as he must act as a blind man. A needle-holder is used merely to introduce the needles; the pushing of them through, as well as all the rest of the operation, must be performed by the fingers alone. The direction direction of the stitches is from behind forwards, also from before backwards, and from the sides to the centre. The greatest caution is necessary, that the needles do not go too far, and catch upon the vagina, or puncture one of the arteries, or the great veins behind the coat of the vagina. To prevent this, the operator must sacrifice his fingers; and the point of the needle, when through, must be bent immediately with the point of the finger, and seized, and drawn through with a pair of forceps. This cannot be done without pricking the fingers; and it might have been thought that the fingers could not escape being dangerously infected, having to work so long afterwards in the morbid ichor. Professor Osiander, however, was never infected; for, immediately after the operation, he always washes his hands repeatedly with soap, and then washes out the pricked wounds with diluted sal volatile, and last of all sucks them out, without applying any suppurants to the wounds. After five or six days, the wounds healed without any inconvenience.

"A waxed thread four times doubled must be drawn through each needle; very often two of these will suffice to draw down the uterus into the vagina; at other times, four are required.

"Many physicians have an erroneous idea of this operation; they believe that the uterus must be drawn out before the body, that is to say, must be brought out entirely. It is equally erroneous to suppose that it is intended that the whole uterus should be cut out, and for this reason the operation for cancer of the womb is by some denied or rejected as impossible.

"By the threads the whole womb is only fixed in the bottom of the vagina to be cut off, but the drawing of it down is sometimes rendered difficult, from an adhesion of the external mouth of the womb to the omentum. Upon a late occasion, from this cause, the uterus could not be drawn down by the threads, and the threads were accidentally cut in introducing the bistoury. Professor Osiander took a pair of lithotomy forceps, laid hold of the orifice of the womb with them, and cut the cervix off.

"The cancerous and scirrhous portion is to be cut off only as far as the healthy substance, which is known by feeling the smooth surface, and elastic firmness of the latter, which are very different from the rough and woody-like scirrhus.

"The crooked bistoury must be small, strong, and sharp, and rounded at the point. It is to be carried up close to the cervix, as high as possible, while an assistant keeps the labia separated. The incision must be made in curve lines, first boldly, and then cautiously, for fear of hurting the vagina. This is Professor Osiander's first and oldest method of operating.

"The second is as follows: When the cervix is already for the most part destroyed by the cancerous fungus, much enlarged, and the cavity full of knotty carcinomatous fungus, and it is neither possible to lay hold of the uterus with the needles, nor draw it down, then he places the patient almost in a horizontal position; makes an assistant lay his hand on the region of the *fundus uteri*, to press down the womb; fixes the bottom of the womb in the hollow of the os sacrum with the fore-finger of the left hand; introduces the middle and ring-finger into the womb, and, with the help of these fingers to guide the cut of the scissars, he extirpates, in small pieces, all the fungous, rough, and scirrhous parts, with a pair of bent-bladed scissars, and an instrument of his own invention. As soon as this is done, he fills up the cavity with a sponge dipped in wine, and in the astringent powder spoken of before, and treats the wound in the manner already mentioned.

"According to the testimony of all those who have submitted to it, this operation is not nearly so painful as one might imagine, and it heals much sooner than could be expected. Nature seems in no part of the human body to be more active in reproducing what is lost, and in healing what is wounded, than in the parts of generation in both sexes. It is surprising to see (for example) a scrotum lost by a mortification, restored again in four weeks. It is no less surprising, for a kind of mouth of the womb to be regenerated in a few weeks after the *cervix uteri* has been totally cut off; or regular menstruation to be re-established equally soon from the half womb left after the operation.

"The duration of the cure is very different, as it is in every operation for cancer. One circumstance, and the result of experience, is of great importance, namely, that neither during the operation, which is conducted with so great difficulty, nor during the subsequent treatment, has one patient hitherto died. Some of them died more than a year afterwards from other causes, as from apoplexia nervosa, dropsy, and the like, or the disease returned suddenly again from new causes, and became incurable; others continued three years and more in good health.

"The sooner a patient resolves to submit to the operation, the longer will she enjoy her recovery; and the better she observes the diet that is prescribed afterwards, the better chance will she have of being freed from the evil for ever.

"It is consolatory to observe, that, in a disease which has been thought incurable, more has been done than was formerly thought possible, and that thus a new path is opened for the art of surgery, by which suffering humanity may derive help and comfort. Let, then, practitioners labour to acquire the courage and dexterity which is required to undertake such an operation, and conduct it to a fortunate termination."

*Remarks by the Editor of the London Journal.*—We have long suspected these diseases to arise from mere inflammation: first, because they are more common at that period of life when inflammation in different organs affect the sex, unless prevented by occasional hæmorrhage from the uterus, or by venesection; next, because at whatever age they may occur, a violent paroxysm or pain frequently precedes, and is relieved by, hæmorrhage. This relief by uterine hæmorrhage is not confined to inflammation in that organ; it often attends the eruptive fever in the exanthemata, and, there is reason to believe, in many other fevers, though it it is too often overlooked or confounded with the periodical discharge, or even considered as a diseased symptom; yet the relief derived from it after parturition, which is now well understood to be attended with inflammation, might induce us to think otherwise.

If inflammation in the uterus continues long, and exceeds certain bounds, we cannot wonder that suppuration or ulceration follows. The nature of the ulceration may be imputed to the structure and secretory property of the uterus, which may differ from other parts under suppuration, as we know its texture is different, and as we see a difference in the sequela of inflammation in various parts according to their structure. But, what has most confirmed our opinion, that the whole depends on inflammation is, that for some years past we have found dreadful pains in the loins and in the whole uterine region, with frequent hæmorrhage, often too at a suspicious period of life, which we apprehended might end in malignant ulcer of the uterus, always relieved by very free and very frequent cupping near the sacrum. Whether these cases would have proved ultimately the disease they were apprehended to threaten, it is impossible to determine, but this we can assert, that all the malignant ulcers, whose history we have been able to trace, have commenced with these symptoms, some of which have continued for years before ulceration. We cannot, therefore, scruple to recommend that practice in the early stage of these complaints, in the more advanced, we submit to our readers the prospect of relief held out by Professor Osiander.

---

Our readers will recollect a paper by Sir Everard Home, "On the Formation of Fat in the Intestines of Living Animals,"* extracted from the Philosophical Transactions. As we profess to glean whatever may relate to pathology, we conceived the authority of the Royal Society, whose ordeal is considered somewhat severe, a sufficient sanction for a naked transcript of the experiments of their vice-president. As the only apology for our unguarded haste, we now offer the following remarks on Sir E. H.'s paper, unaccompanied, like the former, with any observations of our own.

*Observations on Sir Everard Home's Theory of the Formation of Fat;* by William Wadd, Esq. (extracted from his Remarks on Corpulence.)

"Secretion, like every other operation in the animal economy, must for ever be involved in obscurity. How, from the same animal fluid, bile should be secreted by one organ, tears by another, muscular fibre by a third, and osseous substance by a fourth, has excited surprize, and the secretion, as it was hitherto supposed of fat, not less than the rest. This last mystery has at length been solved. A vice-president of the Royal Society assures us, that fat has nothing in common with the secretions, and that he has found

---

* See vol. xxxi. pages 43, and seq.

the the whole manufactory and depot in the intestines. This is proved by a variety of illustrations, which the reader of taste will find in the Transactions of that learned body, vol. ciii. page 146.

"The author of the Pursuits of Literature, remarks, that philosophy is a very pleasant thing, and has various uses; one (by no means the least important) is that it makes us laugh, a well known recipe for making us fat. It is probably, on this account, that most of the scientific bodies in Europe have been attentive to exciting an action, which is said to be peculiar to the human race, and the effect of which we have seen has exalted individuals above the rest of the species. The Royal Society of London, after neglecting this laughter-making property of philosophy for some years, seems, in this instance, inclined to revive it.

"Lest it should be suspected that I have misrepresented the important paper above alluded to, and its accompanying *specimen*, I shall offer a slight analysis of the first; the latter has been analyzed by a chemist, not less celebrated for his accuracy than his modesty, of whom it need only be said that he is the very able successor of Davy at the Royal Institution.

"Sir Everard Home, in examining the *cœca* of different animals, was struck with a thought, which had escaped Mr. Hunter in his investigations on the digestive organs; namely, that fat is formed by a process in the lower intestines, where a "secondary kind of nourishment is extracted from the food." He afterwards tells us, from facts entirely within his own knowledge, (but not different from what was known twenty years before), how adipocere may be formed from animal matter, placed as he describes it on the banks of a sewer. Of this he has specimens in his own possession. Ambergris, he continues, is found only in diseased whales, and in the human intestines *scybalæ* are sometimes found, in 'all respects similar to ambergris.'

"Dr. Babington, with great composure, assures Sir Everard, that a lady who swallowed olive oil, passed some of it, in a state 'to bear being cut by a knife.'

"Another case follows from the same physician; but, as it is only a private communication without Dr. Babington's signature, the young lady's name shall not be mentioned here; though it is inserted in the Transactions, where we read, 'subject to occasional gripings,—at uncertain intervals she voids an oily substance, sometimes mixed with fæces; a specimen of which, procured under circumstances which precluded all possibility of deception, is laid on the table of the Royal Society.'

"It would be tedious to detain the reader with the experiment on the poor duck, or the contents of this cæcum, after his rectum, as it appears, was tied up for a week. But it is impossible to pass over the following instance of dexterity, or to do justice to the author without transcribing his words. A gouty old gentleman had been six days confined to his bed without any evacuation from the bowels. 'I did not let slip,' says Sir Everard, 'the opportunity of his having a very costive stool, deeply tinged with bile, to make the the experiment.' To detail the result might neither be agreeable nor interesting; the film which appeared at the top of the water, after long maceration, required not the experiments of my friend Mr. Brande, to convince us that such a film was oily. After several similar illustrations, Sir Everard concludes,—'On the present occasion, I hope I have collected a sufficient body of evidence to prove that fat is formed in the intestines, and from thence received into the circulation, and deposited in almost every part of the body.'

"Notwithstanding this body of evidence, however, some may say, if fat is received into the circulation, it must be mixed and assimilated with the blood, and, to be afterwards deposited in various parts, it must be again separated. Now, separation is only another word for secretion. This would lead us to believe, that fat has much in common with the secretions; and we might even suspect, that there could be no necessity for this previous manufactory and deposition, were it not for a sentence in the exordium of the paper. 'The more I canvassed this new opinion,' says Sir Everard, 'the greater number of circumstances in favor of it occurred to me; one of the strongest of which is, that there is no other mode I (Sir Everard) am acquainted with, by which animal fat can be formed.'

"However highly we may prize the above Essay, yet its claim to originality may be disputed. Boerhaave has a paper, 'de utilitate explorandorum excrementorum in ægris, &c.' and it is well known by those who have perused the introduction to his edition of Aloisuis Lusitanus, how important he conceived the fat, and how necessary it was to dissolve every part of it, even in the cancellous part of the bones. It does not indeed appear that he offered any *specimena excrementitia* to his hearers, or that he was aware of the value of a gouty old gentleman in the illustration of his Thesis.

"I cannot conclude this account, without fearing my readers may suspect a hoax. Some of them may recollect the celebrated dissertation on a broomstick, and, associating the taste of its learned writer with this paper, may consider it the production of Swift. I must acknowledge that when it was shewn me, as connected with my enquiries, the facetious Dean, and Sir John Hill, instantly occurred to me; a reference to dates, however, will settle the doubts of the most incredulous."

